# Untangling Natural Seascape Variation from Marine Reserve Effects Using a Landscape Approach

**DOI:** 10.1371/journal.pone.0012327

**Published:** 2010-08-20

**Authors:** Brittany E. Huntington, Mandy Karnauskas, Elizabeth A. Babcock, Diego Lirman

**Affiliations:** Marine Biology and Fisheries, Rosenstiel School of Marine and Atmospheric Science, University of Miami, Miami, Florida, United States of America; Smithsonian's National Zoological Park, United States of America

## Abstract

Distinguishing management effects from the inherent variability in a system is a key consideration in assessing reserve efficacy. Here, we demonstrate how seascape heterogeneity, defined as the spatial configuration and composition of coral reef habitats, can mask our ability to discern reserve effects. We then test the application of a landscape approach, utilizing advances in benthic habitat mapping and GIS techniques, to quantify this heterogeneity and alleviate the confounding influence during reserve assessment. Seascape metrics were quantified at multiple spatial scales using a combination of spatial image analysis and in situ surveys at 87 patch reef sites in Glover's Reef Lagoon, Belize, within and outside a marine reserve enforced since 1998. Patch reef sites were then clustered into classes sharing similar seascape attributes using metrics that correlated significantly to observed variations in both fish and coral communities. When the efficacy of the marine reserve was assessed without including landscape attributes, no reserve effects were detected in the diversity and abundance of fish and coral communities, despite 10 years of management protection. However, grouping sites based on landscape attributes revealed significant reserve effects between site classes. Fish had higher total biomass (1.5×) and commercially important biomass (1.75×) inside the reserve and coral cover was 1.8 times greater inside the reserve, though direction and degree of response varied by seascape class. Our findings show that the application of a landscape classification approach vastly improves our ability to evaluate the efficacy of marine reserves by controlling for confounding effects of seascape heterogeneity and suggests that landscape heterogeneity should be considered in future reserve design.

## Introduction

No-take marine reserves have been increasingly promoted as a management tool to conserve biodiversity and prevent over-exploitation of marine communities [1], [2]. Assessing whether reserves meet these objectives relies upon sampling designs that can evaluate management impacts on the communities targeted by reserve designation while controlling for the confounding spatial and temporal effects that could influence the assessment [3]. Yet, the most commonly used analyses for reserve assessment leave results open to interpretation, stressing the need for improved designs to document reserve effects [3], [4]. Existing reserve assessments have been consistently criticized for a myriad of insufficiencies, including limited sample replication [5], non-random reserve placement [6], and inadequate controls for temporal and spatial variability in the systems being protected [7], [8]. The Before-After-Control-Impact (BACI) assessment and its relatives (e.g., BACIPS, Beyond BACI) were developed in response to these criticisms as sampling designs capable of controlling for natural temporal changes [8], [9]. However, all BACI approaches rely on ‘Before’ data collected at the reserve inception; data that are not available for the vast majority of marine reserves [5].

Given the paucity of baseline data, Control-Impact (CI) comparisons are the most commonly used marine reserve assessment methodology, in which control sites outside of the reserve are compared to impact sites within [9]. CI comparisons putatively attribute observed differences to a reserve effect; however, this methodology cannot distinguish between management effects and intrinsic seascape heterogeneity between control and impact sites [3], [7]. Even in well-replicated studies with high numbers of control sites, separating the effects of spatial seascape variation from those of protection can be difficult given that a procedural framework is lacking for selecting appropriate control sites within a heterogeneous seascape [10]. To date, no sampling designs have explicitly quantified and controlled for seascape heterogeneity, defined as habitat configuration and composition, when conducting CI assessments. In a literature review of 68 studies assessing the prevalence of BACI and CI approaches from 2004-2009, only 10 studies (15%) employed a BACI approach. The remaining studies relied on CI assessments. Of these, only 4 (7%) quantified any spatial metric pertaining to seascape measures of habitat configuration or composition when selecting control sites for reserve evaluation.

In both terrestrial and aquatic systems, the response of organisms to heterogeneity in a landscape varies across spatial scales [11]–[13]. Coral reef habitats are no exception. Reef systems are heterogeneous, composed of patches that vary in size, shape and spatial arrangement across the seascape. This spatial context of a patch of reef habitat within the surrounding seascape can exert a strong influence on abundance and distributions of reef-associated organisms, including reef fishes that are commonly targeted for reserve protection [14]–[16]. Hence, marine reserves that span heterogeneous seascapes should take into account this variability when assessing the efficacy of marine reserves to protect reef fish and other marine organisms.

In comparison to the numerous terrestrial-based landscape studies, a landscape ecology approach in marine systems is still in its infancy [17]. Advances in remote sensing and mapping technology have recently enabled marine scientists to quantify submersed seascapes and apply terrestrial landscape metrics to investigate ecological patterns and relationships on spatial scales relevant to marine organisms [18]–[20]. We continue in that vein by applying a multi-scale landscape approach to distinguish between the effects of natural seascape variation and management actions when assessing the impacts of marine reserve designation. This approach is centered on determining the importance of specific seascape configuration and composition metrics on communities targeted for reserve protection. For this investigation, two target communities, reef fish and corals, were identified in our study site of Glover's Reef Marine Reserve, Belize. We examined reserve efficacy to increase biodiversity and biomass of fishes, as well as enhance diversity and cover the coral community through cascading effects that reduce macroalgal cover, a major coral competitor [21]. We describe steps to: (1) quantify seascape spatial heterogeneity of patch reef sites; (2) identify key spatial, compositional, and structural seascape characteristics of patch reefs that correlate to observed variability in both reef fish and coral communities; (3) classify patch reef sites into groups sharing similar seascape attributes; and (4) evaluate reserve efficacy with and without site groupings to compare our ability to discern reserve effects when controlling for seascape variability.

## Methods

### Study Area

**Figure 1:**
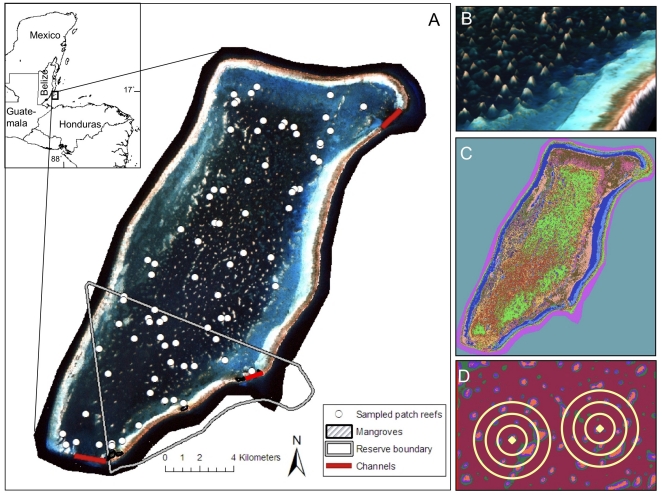
Remote sensing imagery of sampling locations and benthic habitats for Glover's Atoll, Belize. (A) IKONOS satellite imagery of Glover's Atoll showing patch reef study site (white circles). (B) ENVI bathymetric map of NE section of lagoon. (C) Habitat classification map featuring 12 benthic habitat classes. (D) Delineation of 200 m, 500 m, and 1 km buffers around patch reef sites to generate patch density metrics.

Glover's Reef Atoll (87° 48′ W, 16° 50′ N) is located 30 km offshore of Belize, Central America, and comprises an area of 560 km^2^ (Fig. 1A). The atoll perimeter consists of emergent crest reef interrupted by three channel passes. The interior lagoon slopes gently to a depth of 6–18 m and is dotted with approximately 850 patch reefs varying in size from 20 m^2^ to 10,000 m^2^. These patches are primarily elliptical in shape and rise from the lagoon floor to within 0–3 m of the surface. A no-take marine reserve, enforced by wardens since 1998, is located in the southern section of the atoll.

Patch reefs served as the focal habitat for this analysis. Several features of the patch-reef array at Glover's Atoll make this reserve an ideal model system to test the applicability of landscape ecology approach to marine reserve evaluation. First, patch-reef complexes are pervasive, often containing hundreds of individual patches enabling ample replication within the reef system. Second, the discrete boundaries of patch reefs, often surrounded by sand or seagrass, enables spatial metrics of patch composition and configuration to be readily quantified through remote sensing and spatial analyses. Third, due to the geographic isolation and deep waters (>400 m) surrounding the atoll [22], the confounding influence of fish immigration and emigration to and from the atoll are likely limited [23], [24]. Fourth, the size of the atoll limits the maximum distance between reserve and control sites to less than 10 km, suggesting that dispersal ranges of our sampled populations are not limiting [25].

#### Sampling fish and corals

We assessed the fish and benthic communities at 87 submerged patch reefs in 2008–09 using a spatially explicit stratified random sampling design in which the entire lagoon area was divided into 23, equally sized blocks. A random point generator in ArcGIS was used to select a minimum of 3 patch reef sites within each block. A total of 56 non-reserve sites and 31 reserve sites were sampled in three field efforts: May 2008, February 2009 and April 2009. To investigate possible temporal changes over the 10-month sampling period, fish and coral surveys were repeated at 15 randomly selected patches from the total 87. No significant differences in coral cover, coral diversity, fish abundance or fish diversity were detected in this subset from 2008 sampling to 2009, and we therefore pooled the two years of data. Fish abundances were determined using the stationary point-survey method [26] for all observed fishes over 5 cm within a 5m-diameter cylinder. A total of 5 surveys, positioned at the 3 m depth contour on N, E, S and W patch edges and patch center, were completed for each patch reef surveyed using SCUBA. Species, number of individuals, and length were estimated for all observed fish. Fish-length estimates (fork length, cm) were calibrated before each sampling period by estimating lengths of fish-shaped objects until estimates were within 10% of true lengths. Fish lengths were converted to biomass for each cylinder using allometric coefficients from Bohnsack and Harper [27] and were averaged per patch. Fish communities were summarized at each patch reef with the following metrics: (1) species richness, defined as the total number of fish species observed within the 5 cylinders per patch reef; (2) total fish biomass, defined as the sum biomass of all species averaged across the 5 cylinders, and (3) commercial fish biomass, defined as sum biomass of all species observed in the fishery catches [28] (Table S1) averaged across the 5 cylinders.

The benthic composition of each patch reef was determined through the use of digital photography. Photographs of the benthos encompassing a reef area of approximately 0.25 m^2^ were taken every 2 m from 0.5 m above the substrate along transects running the long and short reef axes. Depending on the total patch size, 25–100 images were generated per patch. Images were analyzed to species for scleractinian corals (>2 cm min. diameter), to functional group for benthic biota or to substrate class for non-biotic substrates. Using point-intercept methods, 100 random points were scored per image, on 20 randomly selected images per patch using CPCe v3.5 software [29]. From this analysis, we calculated an average % cover per patch for (1) coral cover and (2) coral:macroalgal ratio (not including turfs or crustose coralline algae). The species richness of stony corals per patch was determined by noting species presence/absence during a 10-minute search interval.

#### Quantifying seascape heterogeneity

A comprehensive approach to quantifying spatial heterogeneity in the seascape combined remote sensing, image analysis and in situ classifications across multiple spatial scales. Seascape metrics were selected based on previous studies linking specific features of seascape and habitat variability to fish and/or coral community structure [16], [30], [31] (Table 1). Metrics were assessed at increasing hierarchical spatial scales when possible to explore the most appropriate spatial extent to our diverse target communities [19]. Metrics of patch composition included measures of area and volume to account for the 3-dimensional nature of the aquatic environment [32]. Patch area, perimeter, and edge: area ratio were calculated using Hawth Analysis Tools for ArcGIS (freely available from www.spatialecology.com/htools) on polygons drawn around the patch boundaries using multi-band, high-resolution (4×4 m ground resolution) IKONOS imagery. An adjusted patch area was also calculated using the percentage of sand and seagrass to account for differences in hard substrate on each patch. Patch volumes were obtained from bathymetric maps generated in ENVI v4.7 based on depth and GPS data collected throughout the atoll at 183 points (Fig. 1B).

**Table 1:** Summary statistics for seascape metrics and patch structure variables.

Seascape category	Metric/variable	Measure	Transformation	Min.	Max.	Mean	CV
Configuration	Distance from channel	km	Log10	0.8	13.1	7.3	43.8
	Distance to mangroves	km	Log10	0.1	16.5	7.4	68.0
	Nearest neighbor	m	Log10	4.2	341.9	111.6	75.7
	Reef area in 1 km buffer	m2	Log10	49456.0	1412634.0	311541.5	81.3
	Reef area in 500 m buffer	m2	Log10	10832.0	310756.0	67834.1	78.8
	Reef area in 200 m buffer	m2	Log10	95.0	47177.0	10509.0	91.8
Composition	Area*	m2	Log10	17.0	17660.0	4020.4	104.2
	Area of hard substrate *	m2	Log10	16.0	12856.0	2905.9	98.4
	Perimeter (m) *	m	Log1010	17.0	696.0	212.6	64.0
	Edge: area ratio*	ratio	Box cox	1.0	36.4.0	14.0	59.5
	Estimated volume* †	m3	None	26.0	33342.0	6022.2	110.8
	Volume* Δ	m3	Box cox	10583.6	387968.6	112978	88.5
	Surface areaΔ	m2	None	653.6	71072.9	10991.5	111.1
Patch structure	Fine-scale rugosity	index	None	1.2	2.1	1.5	13.4
	Coarse-scale rugosityΔ	index	None	1.0	1.6	1.1	3.3
	Structural complexity	index	None	1.0	3.0	1.7	54

All metrics and variables were quantified for each sampling site (n = 87).

*metrics included in PCA of ‘patch size’ due to multicollinearity

† calculated as area * mean fine-scale rugosity

Δ estimated from ENVI bathymetric habitat maps

To generate metrics of the spatial distribution of patch reefs across seascape, a benthic habitat map of the lagoon was made using a supervised spectral classification in ERDAS Image Analysis™ for ArcGIS v9.2 (Fig. 1C). Classes delineating patch-reef habitats were merged into a single layer and compared for accuracy to hand-drawn polygons for each patch. Landscape metrics of patch density were calculated using 200, 500 m and 1 km buffers around each reef to explore appropriate spatial extent for fish and coral communities (Fig. 1D). Nearest neighbor distances were determined by creating a center point within each patch reef polygon and calculating the minimum distance between points. Distance-to-habitat features, including mangrove habitats and the two large channel openings were quantified as potential landscape metrics influencing fish community.

Structural complexity of each patch reef was assessed a three different resolution scales. At the patch reef scale, an in situ score of structural complexity was determined based on a ordinal scaling in which 0 indicated no vertical relief, while reefs with exceptionally high complexity were given a rating of 3 [33]. Coarse-scale rugosity was estimated by calculating the maximum patch length and width in ArcMap and assessing the change in depth between consecutive 4×4 m grid cells from the bathymetric maps over the entire length of both diameters. Fine-scale rugosity measures were taken in situ along 5 haphazardly-placed, 10 m transects using a 2 cm-link chain closely draped over the benthic contours.

### Statistical analysis

To investigate which seascape metrics explained the greatest amount of variation in fish and coral community parameters, canonical correspondence analysis (CCA) was used. In CCA, regression analysis is used to find the best possible relationship between multiple environmental variables and multivariate community response data, assuming key environmental variables have been measured and the community response is unimodal in relation to these variables. Multicollinearity between seascape metrics was explored through correlation matrices. When evident (r>0.2), a principle component analysis was conducted on the co-linear metrics and the first principal component was used in subsequent analyses as an independent explanatory variable [34]. Separate CCAs were conducted to describe the relationships among seascape metrics and (1) fish composition (i.e., fish species richness, total biomass, and commercial biomass as defined above), and (2) coral composition (i.e., coral species richness, % cover, and coral:macroalgal ratio). Seascape metrics and community response parameters were log10-transformed (or arcsine square-root transformed for % cover data) as needed before analyses to normalize data and ensure homogeneity of variance. Akaike's information criterion was used to select the simplest multivariate regression model that explained the maximum amount of variation for each community [35], [36]. Significance of the selected model was tested using Monte Carlo Permutation tests.

Separate hierarchical clustering analyses were preformed for coral and fish to classify patches together into ‘seascape groups’ sharing similar attributes of the significant seascape metrics identified for fish and for corals in the CCA analyses. Reserve effects were then evaluated using a modified Control-Impact design, in which reserve effects were only tested among patch reefs sharing the same seascape grouping for fish and corals, respectively. Comparisons of the fish assemblage (e.g. species richness, biomass, and commercially-valued biomass) and coral assemblage (e.g. richness, cover, and coral:macroalgal ratio) between management zones were conducted using one-way analysis of variance (ANOVA). We then repeated our analyses for each response variable using a traditional Control-Impact methodology with all 87 patch reef sites. Reserve effects were then compared between the two Control-Impact assessments.

Following detection of reserve effects, additional analyses were conducted to determine which organisms were influenced by reserve protection. Community similarity within coral and fish communities with respect to reserve protection and patch type were calculated in multidimensional space using a two-way crossed analysis of similarity (ANOSIM). Community similarity matrices were calculated using a Bray-Curtis index on 4^th^ root-transformed abundance data in order to reduce the contribution of common species [37]. To determine if specific functional groups or trophic levels were more response to reserve protection than others, the fish community was classified by target/non-target species, diet, and trophic level. Analyses between reserve effects and fish class or species were then conducted within a given patch reef grouping to identify which organisms were responding to both seascape heterogeneity and reserve protection.

## Results

### Identifying key seascape metrics

**Figure 2:**
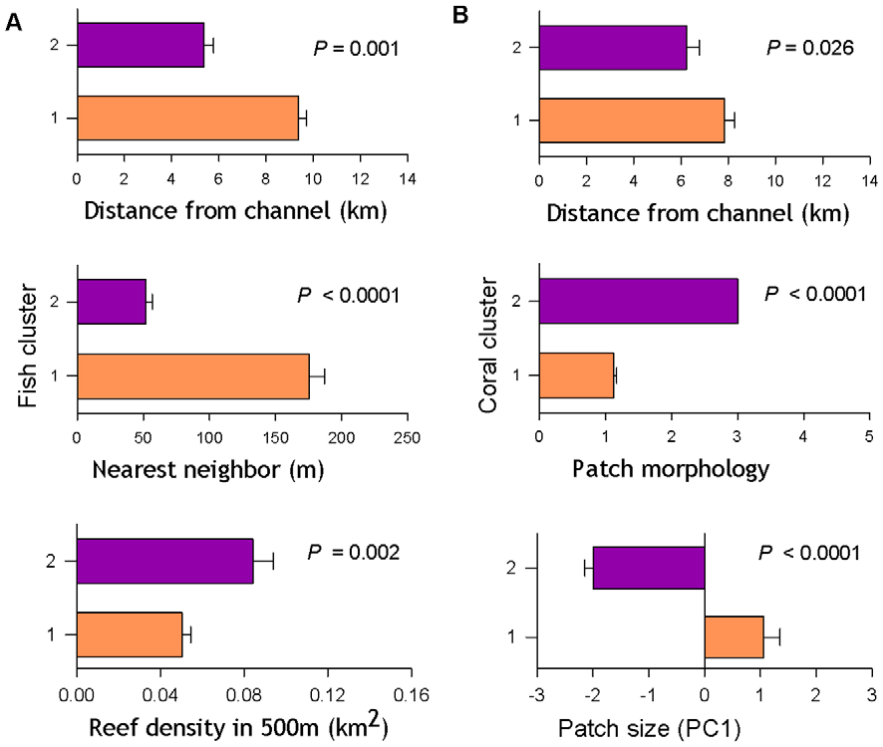
Seascape attributes by patch reef site groups. Attributes of (A) fish and (B) coral site groups for each key seascape metric. Group 1 attributes are in purple; group 2 in orange. One-factor ANOVA significance values for each metric are shown.

Three seascape-level metrics of spatial configuration were identified in CCA analyses as explaining the greatest amount of variation in the fish community: distance from channel, patch reef area within a 500 m buffer, and nearest neighbor distance (Table 2). Using these seascape configuration metrics, patch reefs were clustered into two groups (hereafter called Fish Type I and Fish Type II for simplicity), which was sufficient to generate significant differences between groups for each seascape metric (Fig. 2A; ANOVA; P<0.05) and enabled maximum sample sizes within a group for subsequent analyses of reserve effects. Type I patches are located further from channels, surrounded by a lower amount of patch-reef area within a 500 m buffer, and are more isolated. Type II patches are closer to the channels, have more neighboring patches within 500 m, and are less isolated.

**Table 2:** Summary of best fit model from CCA analysis.

Community	Dependent variables	Significant seascape metrics	Variance explained by 1st axis	AIC	F-statistic	P-value
Fish	Richness	distance from channel				
	Total biomass	reef area in 500 m buffer				
	Commercial biomass	nearest neighbor	0.11	−148.4	3.57	0.008
Coral	Richness	distance from channel				
	% cover	patch size^*^				
	Coral:macroalgae	structural complexity	0.11	−205.6	3.12	0.014

Models selected using AIC value to examine the relationship between seascape metrics and fish community and coral community on study patch reefs (n = 87). Statistical significance of each model is reported using Monte Carlo simulations (999 permutations) to generate an F-statistic and P-value. ^*^ 1^st^ principal component using multicollinear patch size metrics.

CCA analysis was used to identify 3 seascape level metrics that explained the greatest amount of variation in the coral community: distance from channel, ‘patch size’, and structural complexity of the patch (Table 2). ‘Patch size’ was generated using a PCA on 6 multicollinear metrics pertaining to the patch area and using first principal component as a seascape metric (PC1  = 98.4% of total variance; Table 1). Using these three metrics, patch reefs were clustered into two groups (Coral Type I and Coral Type II). Type I patches are further from the channels, larger, and consist of a dome-shaped morphology. Type II patch reefs are closer to the channels, smaller, and have a complex morphology. As was the case for the Fish patches, Coral Type I and Type II patches show significant differences in all three seascape metrics between groups (Fig. 2B; ANOVA; P<0.05).

### Evaluating reserve effects

**Figure 3:**
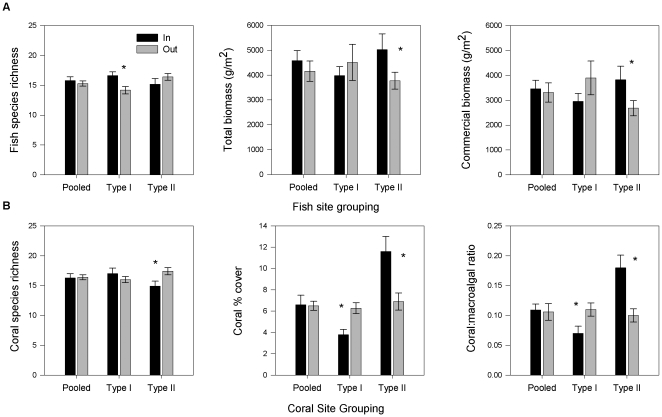
Reserve responses for pooled versus clustered sites. Fish response variables (A) shown in top panels and coral response variables (B) on bottom panels. Significant differences (P<0.05) between in (dark bars) and outside reserve (light bars) are denoted with an *.

We assessed differences in fish species richness, total biomass and commercially important biomass inside and outside of reserve using two different site grouping approaches. No significant reserve effects were detected for any fish community response variable when seascape differences among patch-reef sites were disregarded (Table 3; Table S2). However, grouping sites based on key seascape metrics identified using multivariate ordination models made it possible to detect significant reserve effects (Fig. 3 and Table 3). Commercial fish biomass was approximately 75% greater inside the reserve than outside for Type II patches (by one-way ANOVA, F_1,43_ = 8.05, P = 0.007). A similar significant increase of 50% was seen in total fish biomass from outside the reserve to inside (F_1,43_ = 7.479, P = 0.009). There was no difference in fish species richness inside versus outside reserve for either site grouping approach (Table S2).

**Table 3:** Reserve effects using different site classification scenarios.

**Community**	**Response variable**	**Pooled (n = 87)**	**Type I (n = 58)**	**Type II (n = 29)**
Fish	Species richness	--	--	--
	Total biomass	--	--	+50%**
	Commercial biomass	--	--	+74%**
		**Pooled (n = 87)**	**Type I (n = 42)**	**Type II (n = 45)**
Coral	Species richness	--	--	-17%*
	% cover	--	-65%**	+68%**
	Coral: macroalgal	--	-57%*	+80%**

Proportional difference for each fish and coral response variable under varying clustering scenarios between reserve and non-reserve sites. Only significant results are shown. Positive values are greater inside reserve versus outside; negative values are lower inside reserve versus outside.

*P<0.05 and

**P<0.01 as determined using one-way ANOVA comparing reserve and non-reserve sites.

As with the fish community, no significant differences between reserve and nonreserve sites were detected in coral community parameters when all patch reef sites were pooled (Table 3; Table S2). However, grouping sites that shared similar attributes of relevant seascape metrics to the coral community revealed significant reserve effects in all three community parameters (Fig. 3). Coral Type II patch reefs responded positively to reserve protection, increasing in both coral cover and coral:macroalgal ratio for reserve sites. Coral cover in Type II patches was 68% higher inside versus outside the reserve (F_1,27_ = 8.24, P = 0.008). Similarly, coral:macroalgal ratio increased 80% for Type II clustered sites within the reserve versus outside (F_1,27_ = 14.22, P<0.001).

Yet, reserve effects were not uniform across or within site grouping for either the fish or coral communities (Table 3). Results for Coral Type I patches showed negative reserve responses for 2 of the 3 response variables. For this patch group, coral cover and coral:macroalgal ratio was lower inside the reserve (coral cover: one-way ANOVA, F_1,56_ = 9.037, P = 0.004; coral:macroalgal: one-way ANOVA, F_1,56_ = 5.362, P = 0.024). Similarly, Fish Type II patches showed positive responses, while Type I patches showed no differences between reserve and nonreserve sites (Table 3). Coral Type II reefs, despite responding positively in coral cover and coral:macroalgal ratio to reserve protection, did show a small but significant decline in mean coral species richness from 17.4 (±0.61) species outside the reserve to 14.9 (±0.84) species inside the reserve (Fig. 3).

**Figure 4:**
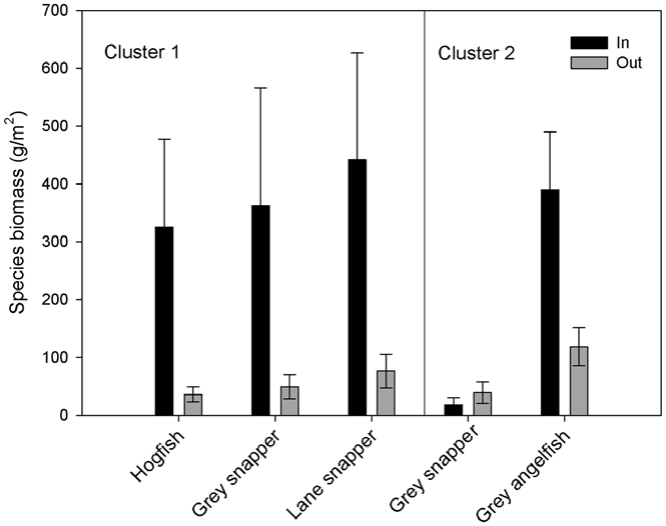
Species-specific fish reserve response by patch reef seascape grouping. Only species showing statistically significant (P<0.05) differences between in (dark bars) and non-reserve (light bars) sites within a patch reef type are shown. The left panel shows fish species from Patch Type I; the right panel shows fish species from Patch Type II.

Analysis of similarity (ANOSIM) results revealed that coral communities were statistically indistinguishable between both Type I and Type II patches and across the reserve boundary (P>0.05). ANOSIM of the fish community revealed significant differences by patch type and reserve protection, but only Patch Type II reefs showed separation of community composition across the reserve boundary (P = 0.001, R = 0.35). Non-commercial fish species showed no significant response to reserve protection within either Type I or Type II patch reefs (one-way ANOVA, P>0.05), suggesting that the positive reserve effect detected among Type II patch reef was driven by commercially important fish species sensitive to seascape heterogeneity and reserve management. Further investigation of the differences in commercial fish species composition on Type II patches showed no significant difference across the reserve boundary based on fish diet or trophic level (one-way ANOVA, P>0.05). Species-specific responses within Type II patches revealed significantly greater biomass within the reserve for 3 species; two snappers (Lutjanus griseus and L. synagris) and the hogfish (Lachnolaimus maximus; Figure 4; one-way ANOVA, P<0.05). In contrast, Type I patches revealed significant reserve responses for the grey angelfish (Pomacanthus arcuatus) and the grey snapper (Lutjanus griseus); L. griseus was more abundant outside the reserve boundary while P. arcuatus was more abundant within the reserve on Type I patches (Figure 4; one-way ANOVA, P<0.05).

## Discussion

Coral reef ecosystems are complex, three-dimensional seascapes that exhibit multi-scaled structural heterogeneity. We hypothesized that this seascape heterogeneity would confound our ability to detect reserve effects using existing Control-Impact assessment strategies. This was found to be the case at Glover's Atoll Marine Reserve, Belize, where we have shown that it is possible to detect significant differences between reserve and nonreserve sites by separating out key aspects of the spatial variability in the system. Our findings agree with those from terrestrial [12], [38] and marine [19], [39] investigations, in which specific landscape features, quantified over spatial scales exceeding the local scale, were associated with particular group of organisms.

It is not surprising to report that reserve effects vary across the seascape. What is surprising is that current methods for assessing reserve impacts, in the absence of baseline data, do not account for this variability. The composition and placement of individual patch reefs within the seascape has been shown to exert a strong influence on the assemblage structure of reef fishes [16], [39], [40]. We are not aware of any studies investigating the response of coral assemblages to landscape-scale metrics prior to our investigation, but it is reasonable to presume that corals would also be responsive to seascape-level heterogeneity. Therefore, to accurately assess the efficacy of marine reserves targeting organisms such as fish and coral communities, a methodology that integrates habitat variability at the appropriate ecological scales is necessary [18]. Our approach sets forth a new protocol for controlling for seascape differences that can be both readily assessed and used to pair reserve site to appropriate control sites for Control-Impact assessment.

Our results corroborate those of Friedlander et al. [18] who concluded that habitat type was an important predictor of the effectiveness of marine reserves in Hawaii. Similarly, Harborne et al. [41] found that robust reserve effects for a Caribbean coral reef reserve were restricted to a specific habitat type, presumably in response to fish habitat preferences. A recent study by Hamilton et al. [42] acknowledged the role of seascape variability at large spatial scales over which marine networks may operate. Similarly to our goals for this study, they grouped reserve and control sites into biogeographic zones based on differences in fish community assemblages across the marine network driven by large-scale abiotic gradients. While both Harborne et al. and Hamilton et al. demonstrate the ability of interhabitat variability to influence the spatial distribution of organisms and thereby potentially confound reserve evaluation, ours is the first study to evaluate the potential of intrahabitat variability, within a single habitat ‘type’ of coral reef, to influence organism distributions and mask reserve effects. This suggests that seascape heterogeneity can be subtle but still informative to guide the selection of appropriate reference sites when estimating reserve effects. Conducting this analysis within the single reef type of shallow-water patch reefs does prevent extrapolating the specific seascape metrics and reserve responses detected in this case study to other reef systems. However, the landscape approach used to identify these seascape metrics and control for them during reserve assessment can be readily applied in a diverse array of marine habitats.

Inferring ecological processes of community assembly based on landscape-scale patterns is not the objective of the approach we have presented in this study. The seascape variables identified for the patch reef grouping in our Glover's Reef case study are not necessarily drivers for the variations observed in the coral and fish communities. Rather seascape metrics, like all metrics of spatial heterogeneity in a landscape framework, serve directly as a means to quantifying variability across the system and indirectly as a proxy for underlying ecological processes [13]. Further analyses can offer a step forward to understanding the mechanistic processes regulating the community composition in this shallow lagoon system. Our analyses suggest that commercial fish species, rather than a particular functional group or trophic level, are driving the positive effects of reserve protection detected on Type II patches. Of these, 3 species, hogfish (Lachnolaimus maximus), grey snapper (Lutjanus griseus) and lane snapper (Lutjanus synagris) appear to drive not only a positive response to reserve protection, but also a response that is sensitive to seascape heterogeneity. While these species showed strong reserve responses (biomass within reserve > biomass outside reserve), this response varied according to patch type.

Fish and coral assemblages showed different relationships to seascape metrics operating at varying spatial scales, suggesting an organism perspective is important. Habitat area and morphology at the patch-scale were a significant factor explaining the observed variation in the diversity and abundance for corals. In contrast, meso-scale (100 s–1000 s m) factors of nearest neighbor and reef area within a 500 m buffer were significant factors explaining composition of fishes. Interestingly, benthic complexity of the patch reefs (i.e. rugosity) at the fine or medium grain scale was not found to be an important predictor for fish or coral assemblage parameters. This suggests that when patterns of community composition are assessed and constrained to a single, topographically complex habitat type, landscape level parameters may be better predictors of marine assemblage structure.

For the large number of marine reserves lacking baseline data, augmenting the traditional Control-Impact reserve assessment with the seascape approach can improve reserve evaluation by controlling for influential aspects of seascape variability that affect target populations. While applied here to shallow water patch reef environments, this approach is repeatable in other marine systems given the increased access to high-resolution benthic habitat maps and GIS technology [18], [20]. Coupling existing habitat maps and free-source satellite imagery with simple image analysis techniques can prove a viable means to creating inexpensive seascape metrics for a diverse array of marine reserve habitats. Additionally, this method can be applied ex post facto to existing reserve assessment data to generate seascape metrics that be used to ensure that appropriate control sites are compared to impact sites to determine reserve efficacy. Lastly, this approach can be tailored to specific organisms targeted by reserve mandates, providing a more exact analysis of reserve effects to the species in question. In summary, this landscape approach provides a cost-effective, improved assessment of management efforts and ultimately, improved conservation for a variety of marine ecosystems.

We stress the need to control for spatial heterogeneity in the evaluation of marine reserves, but application of these landscape ecology principles may improve criteria for reserve placement and design [10], [18]. Reserve effects at Glover's Atoll were not uniform across groups of patch reefs; positive reserve effects were detected in some patch reefs types and negative (or neutral) effects in others. These differential reserve responses correlated with variations in seascape heterogeneity, indicating that reserve placement would benefit from a more nuanced classification of marine habitat types across the seascape. For example, greater meso-scale connectivity between patches, measured as patch density and nearest neighbor distance, was important to supporting more diverse and abundant fish community parameters in this shallow patch reef system. Hence, reserve expansion at Glover's Atoll should target patch reefs arrays that share these spatial configuration attributes, if the management goal is to increase fish diversity and biomass. We see the future of marine reserve design guided by spatial explicit management schemes that incorporate structure, connectivity, and reef context to ensure that protected habitats respond favorably to reserve management.

The establishment of marine reserves as a conservation tool has increased rapidly over the past decade. Yet the absence of baseline data, even within relatively well-replicated studies, makes it challenging to separate management effects from natural variability in populations driven by seascape differences. A weak assessment design that fails to capture reserve effects when they are present can generate false conclusions about reserve efficacy, seriously crippling management efforts to expand the use of marine reserves as a conservation tool. The burden of proof rests on managers and scientists to clarify how marine reserves can function as viable strategies for conservation and population replenishment. Therefore, we need a better understanding of the effects of reserves, which can be positive, negative or mixed. The use of a robust assessment methodology should be implemented to ensure that, when present, positive or negative effects can be properly ascertained. We suggest that the seascape approach applied in this study is one such method, and will serve as a powerful tool to improve our ability to distinguish management effects from natural system variation in future assessments of reserve efficacy.

## Supporting Information

Table S1 Commercially important fish species observed during sampling for Glover's Atoll.
(0.04 MB DOC)

Table S2 ANOVA results of reserve effects on fish and coral response variables using varying site classification scenarios.
(0.05 MB DOC)
