# Supplementary material for: Untangling Natural Seascape Variation from Marine Reserve Effects Using a Landscape Approach
Source: PLoS One. 2010 Aug 20;5(8):e12327. doi: 10.1371/journal.pone.0012327 (PMC2924891; doi:10.1371/journal.pone.0012327)
Supplement: Table S1 — Commercially important fish species observed during sampling for Glover's Atoll. (0.04 MB DOC) [file pone.0012327.s001.doc]

Table S1. Commercially important fish species observed during sampling for Glover’s Atoll.

| **Scientific name** | **Common Name** |
| --- | --- |
| Balistes vetula | Queen triggerfish |
| Calamus calamus | Saucereye porgy |
| Canthidermis sufflamen | Ocean triggerfish |
| Caranx ruber | Bar jack |
| Cephalopholis fulva | Coney |
| Epinephelus cruentatus | Graysby |
| Epinephelus guttatus | Red hind |
| Epinephelus morio | Red grouper |
| Epinephelus striatus | Nassau grouper |
| Gerres cinereus | Yellowfin mojarra |
| Holacanthus ciliaris | Queen angelfish |
| Lachnolaimus maximus | Hogfish |
| Lutjanus analis | Mutton snapper |
| Lutjanus apodus | Schoolmaster snapper |
| Lutjanus griseus | Gray snapper |
| Lutjanus mahagoni | Mahogany snapper |
| Lutjanus synagris | Lane snapper |
| Mycteroperca bonaci | Black grouper |
| Ocyurus chrysurus | Yellowtail snapper |
| Pomacanthus arcuatus | Gray angelfish |
| Pomacanthus paru | French angelfish |
| Pseudupeneus maculatus | Spotted goatfish |
| Scarus vetula | Queen parrotfish |
| Scomberomorus regalis | Cero mackerel |
| Sparisoma aurofrenatum | Redband parrotfish |
| Sparisoma chrysopterum | Redtail parrotfish |
| Sparisoma viride | Stoplight parrotfish |
| Sphyraena barracuda | Barracuda |
