# Supplementary material for: Untangling Natural Seascape Variation from Marine Reserve Effects Using a Landscape Approach
Source: PLoS One. 2010 Aug 20;5(8):e12327. doi: 10.1371/journal.pone.0012327 (PMC2924891; doi:10.1371/journal.pone.0012327)
Supplement: Table S2 — ANOVA results of reserve effects on fish and coral response variables using varying site classification scenarios. (0.05 MB DOC) [file pone.0012327.s002.doc]

Table S2. ANOVA results of reserve effects on fish and coral response variables using varying site classification scenarios.

| **Community** | **Site classification** | **Response** | **df (effect,error)** | **F-ratio** | **P** |
| --- | --- | --- | --- | --- | --- |
| Fish | Pooled | richness | 1, 85 | 0.040 | NS |
|  | (n =87) | total biomass | 1, 85 | 2.039 | NS |
|  |  | commercial biomass | 1, 85 | 1.718 | NS |
|  | Type I | richness | 1, 40 | 0.3621 | NS |
|  | (n = 58) | total biomass | 1, 40 | 0.342 | NS |
|  |  | commercial biomass | 1, 40 | 0.996 | NS |
|  | Type II | richness | 1, 43 | 0.179 | NS |
|  | (n = 29) | total biomass | 1, 43 | 7.479 | 0.009* |
|  |  | commercial biomass | 1, 43 | 8.050 | 0.007* |
| Coral | Pooled | richness | 1, 85 | 0.04 | NS |
|  | (n = 87) | % cover | 1, 85 | 0.135 | NS |
|  |  | coral:macroalgae | 1, 85 | 0.003 | NS |
|  | Type I | richness | 1, 56 | 0.846 | NS |
|  | (n = 42) | % cover | 1, 56 | 9.037 | 0.004* |
|  |  | coral:macroalgae | 1, 56 | 5.362 | 0.024 |
|  | Type II | richness | 1, 27 | 5.171 | 0.031 |
|  | (n =45) | % cover | 1, 27 | 8.243 | 0.008* |
|  |  | coral:macroalgae | 1, 27 | 14.222 | 0.001* |

* denotes highly significant p values (p < 0.01).
